# Morphological and Molecular Characterization of Dietary*-*Induced Pseudo-Albinism during Post-Embryonic Development of *Solea senegalensis* (Kaup, 1858)

**DOI:** 10.1371/journal.pone.0068844

**Published:** 2013-07-16

**Authors:** Maria J. Darias, Karl B. Andree, Anaïs Boglino, Josep Rotllant, José Miguel Cerdá-Reverter, Alicia Estévez, Enric Gisbert

**Affiliations:** 1 Cultius Experimentals, Institut de Recerca i Tecnologia Agroalimentàries, Sant Carles de la Ràpita, Catalunya, Spain; 2 Patobiología Molecular Acuática, Instituto de Investigaciones Marinas, Consejo Superior de Investigaciones Científicas, Vigo, Galicia, Spain; 3 Fisiología de la Reproducción de peces, Instituto de Acuicultura Torre de la Sal, Consejo Superior de Investigaciones Científicas, Castellón, Valencia, Spain; Rutgers University, United States of America

## Abstract

The appearance of the pseudo-albino phenotype was investigated in developing Senegalese sole (*Solea senegalensis,* Kaup 1858) larvae at morphological and molecular levels. In order to induce the development of pseudo-albinos, Senegalese sole larvae were fed Artemia enriched with high levels of arachidonic acid (ARA). The development of their skin pigmentation was compared to that of a control group fed Artemia enriched with a reference commercial product. The relative amount of skin melanophores, xanthophores and iridophores revealed that larval pigmentation developed similarly in both groups. However, results from different relative proportions, allocation patterns, shapes and sizes of skin chromatophores revealed changes in the pigmentation pattern between ARA and control groups from 33 days post hatching onwards. The new populations of chromatophores that should appear at post-metamorphosis were not formed in the ARA group. Further, spatial patterns of distribution between the already present larval xanthophores and melanophores were suggestive of short-range interaction that seemed to be implicated in the degradation of these chromatophores, leading to the appearance of the pseudo-albino phenotype. The expression profile of several key pigmentation-related genes revealed that melanophore development was promoted in pseudo-albinos without a sufficient degree of terminal differentiation, thus preventing melanogenesis. Present results suggest the potential roles of *asip1* and *slc24a5* genes on the down-regulation of *trp1* expression, leading to defects in melanin production. Moreover, gene expression data supports the involvement of *pax3*, *mitf* and *asip1* genes in the developmental disruption of the new post-metamorphic populations of melanophores, xanthophores and iridophores.

## Introduction

Although more than 378 loci (171 cloned and 207 uncloned genes) have been identified as being involved in vertebrate pigmentation [1], the underlying bases of pigment pattern development are far from being completely understood. Nevertheless, some pigmentation-related mechanisms have been described in mammals, which represent a relatively straightforward example of genetic color determination among vertebrates [2]. While mammals have only one class of pigment cell, the melanocytes (which produce variations of black, brown, red or yellow pigment), fishes constitute one of the most colorful vertebrates, where color can be determined by up to six different types of chromatophores: melanophores (black), xanthophores (yellow), erythrophores (red), iridophores (iridescent, blue, silver or gold), leucophores (dull, whitish) and cyanophores (blue) [3]. Together, these cells can produce almost any spectacular color combination that can be seen, for instance, in the community of fish from a coral reef. In spite of these added complexities, many of the same genes and control networks found in mammals are conserved in fish [4], [5]; and considering their small size and easy manipulation, fish are suitable models for a better understanding of vertebrate pigmentation. Indeed, fish have been used as models for melanoma research because it has been shown tissues within fish share molecular signatures and histopathological features with human cancers [6]. The genetics of pigmentation have been explored in several model teleost fish including zebrafish [7], [8], medaka [9], fugu [10], goldfish [11], [12] and, recently, in flatfish [13], [14], [15]. Flatfish are particularly useful to analyze the origin of pigmentation disorders during the ontogeny because altered pigmentation can be induced under intensive rearing conditions [16], [17], [18], [19]. Senegalese sole is a flatfish species known to develop pseudo-albinism when fed high levels of dietary arachidonic acid (ARA) during their development [17]. However, to our knowledge, there is no information about the possible mechanism that underlies this process. The process of pigmentation development can be seen as a cooperative relationship among three different processes: tissue remodeling (involving apoptosis), cellular differentiation of chromatophores, and pigment production. As a previous step, we have recently studied in the ocular side of this species the morphological and molecular ontogeny of skin pigmentation [15], which are essential to elucidate the mechanisms of formation of the adult pigmentation pattern and to understand when and how the albino phenotype appears. Gene markers for the above mentioned processes were seen to alter in a progression that was in synchrony with metamorphosis. The above cited study revealed different stages of skin pigmentation and development in Senegalese sole that coincided with the progress of metamorphosis and patterns of gene expression: i) pre-metamorphosis period (2–11 dph), low expression of a marker of apoptosis (*casp3*) and genes related to melanogenesis and high expression of melanophore differentiating genes; ii) pro-metamorphosis period (11–19 dph), high expression of *casp3* (apoptosis and tissue remodeling) and melanophore differentiating and melanogenic genes; iii) post-metamorphosis (19–47 dph), low expression of all analyzed genes, especially those associated to melanophore differentiation. Major molecular changes in the pigment pattern occurred during pro-metamorphosis and morphological changes in the population of melanophores, xanthophores and iridophores were evidenced at post-metamorphosis to enable the juveniles to conform to the adult pattern of pigmentation [15].

In this report we investigated the morphological development of pseudo-albinism in Senegalese sole and the quantitative expression of eleven pigmentation-related genes to find out if any transcriptional modulation could explain the deviation from normal patterns of pigmentation. In order to obtain the pseudo-albino phenotype, fish larvae were fed during their development with live prey enriched with high levels of ARA, a powerful inhibitor of pigmentation [17]. The pseudo-albino phenotype in Senegalese sole was the result of a disruption of the signaling for the dorsal-ventral patterning during metamorphosis and was characterized by the presence of the pigment cells that differentiated during embryogenesis [20] and the very early stages of larval development (genetically programmed chromatoblast differentiation) and by the absence of pigment cells that should be formed after metamorphosis for development of the adult pigmentation pattern (environmentally modifiable chromatoblast differentiation). Gene expression results provided evidence that *pax3*, *mitf* and *asip* were involved in the developmental disruption of the new post-metamorphic populations of melanophores, xanthophores and iridophores and that melanogenesis was disrupted through the negative regulatory action of *asip1* and *slc24a5* on *trp1* gene expression.

## Results

### Growth, Survival, Metamorphosis and Pigmentation Success

No differences in growth and survival rates were found at the stage of post-embryonic development for both groups in Senegalese sole larvae (control and ARA) (Table 1). The eye migration index, which is used to follow the progress of metamorphosis in flatfish [21], was similar in both dietary treatments. However, at the end of the experiment, the control group was composed of 99% pigmented larvae, whereas 80% of the larvae from the ARA group became pseudo-albinos (Table 1).

**Table 1:** Larval size in dry weight (DW, mg), standard length (SL, mm), specific growth rate (SGR, % day^−1^), survival rate (%), pigmentation (%) and eye migration index (I_EM_) of Senegalese sole fed the two different dietary treatments.

	AGE	CONTROL	ARA
**DW**	15 dph	0.09±0.01	0.09±0.01
	30 dph	0.77±0.07	0.89±0.06
	50 dph	1.92±0.24	1.71±0.32
**SL**	15 dph	4.18±0.05	4.35±0.05
	30 dph	6.401±0.07	6.75±0.07
	50 dph	8.75±0.22	8.71±0.17
**SGR**	2–50 dph	0.08±0.00	0.08±0.00
**Survival**	50 dph	97.3±0.25	98.4±0.24
**Pigmentation**	50 dph	99.1±0.50^a^	18.6±12.9^b^
**Metamorphosis**	15 dph	2.97±0.06	3.04±0.10
	30 dph	5.94±0.39	5.86±0.24
	50 dph	5.94±0.03	5.96±0.03

Initial (2 dph) DW and SL of larvae were 3.07±0.02 mm and 36.7±1.6 µg, respectively. Values are expressed as mean ± SEM (N = 4). Superscript letters indicate significant differences among dietary treatments (One-way ANOVA, *P*<0.05).

### Morphological Development of Pseudo-albino Phenotype

**Figure 1:**
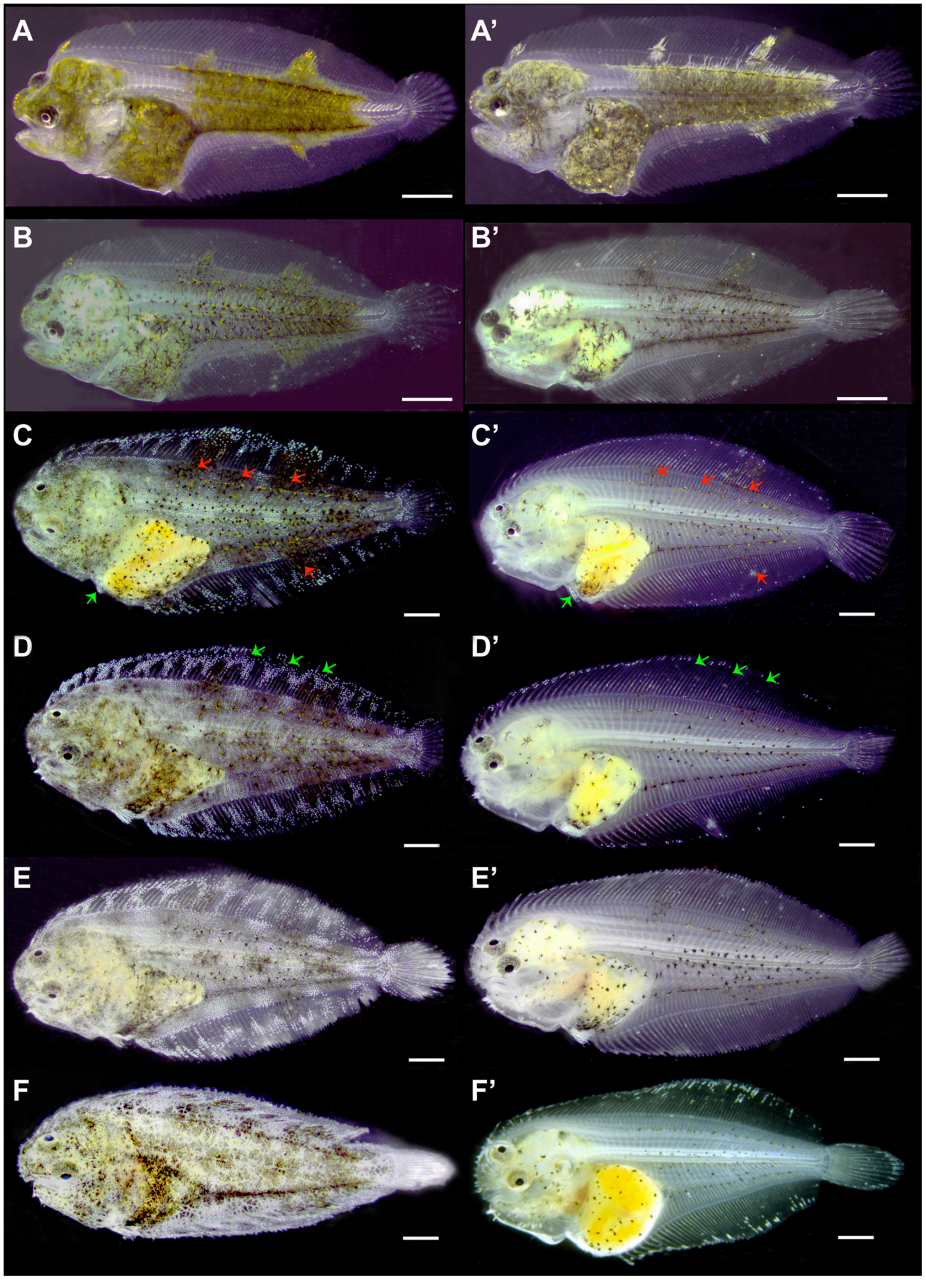
Morphological comparison of the ontogeny of skin pigmentation in Senegalese sole larvae from the control (A–F) and ARA (A’–F’) groups. A–A’) 22 dph, B–B’) 27 dph, C–C’) 33 dph, D–D’) 35 dph, E–E’) 41 dph, F–F’) 47 dph. At 22 dph, the pigmentation pattern of larvae from both dietary groups is similar, showing a very dense net of melanophores, xanthophores and leucophores that covers the head, the digestive cavity and the intermediate region of the trunk. They have also two patches of chromatophores in the dorsal fin and another one in the anal fin. At 27 dph, although the relative amounts of melanophores, xanthophores and iridophores was similar in both groups (Fig. 2), the skin of larvae from the ARA group appeared less pigmented than that of the larvae from the control group due to the progressive disappearance of leucophores that confers the greenish aspect to the skin. At 33 dph, the amount of melanophores in control larvae was higher than in those fed with ARA. Red arrows indicates the allocation of patches of melanophores, xanthophores and leucophores in pigmented individuals (C) and their absence in future pseudo-albino individuals (C’). Green arrows show the presence of iridophores in the pelvic fins of specimens from both groups. At 35 dph the pseudo-albino phenotype is already established. The differences in the amounts of xanthophores and iridophores between pigmented (D) and pseudo-albino (D’) specimens became more evident (Fig. X). Green arrows show aggrupation of iridophores in the dorsal fin in pigmented individuals (D) and their absence in future pseudo-albino individulas (D’). From 35 to 47 dph, the amount of chromatophores in pseudo-albinos remained invariable. However, because there was an increase in the amount of melanophores and xanthophores in pigmented individuales from 41 to 47 dph there was a significant difference in the amount of all chromatophores between pigmented and pseudo-albino individuals at 47 dph. Scale bar, 600 µm.

**Figure 2:**
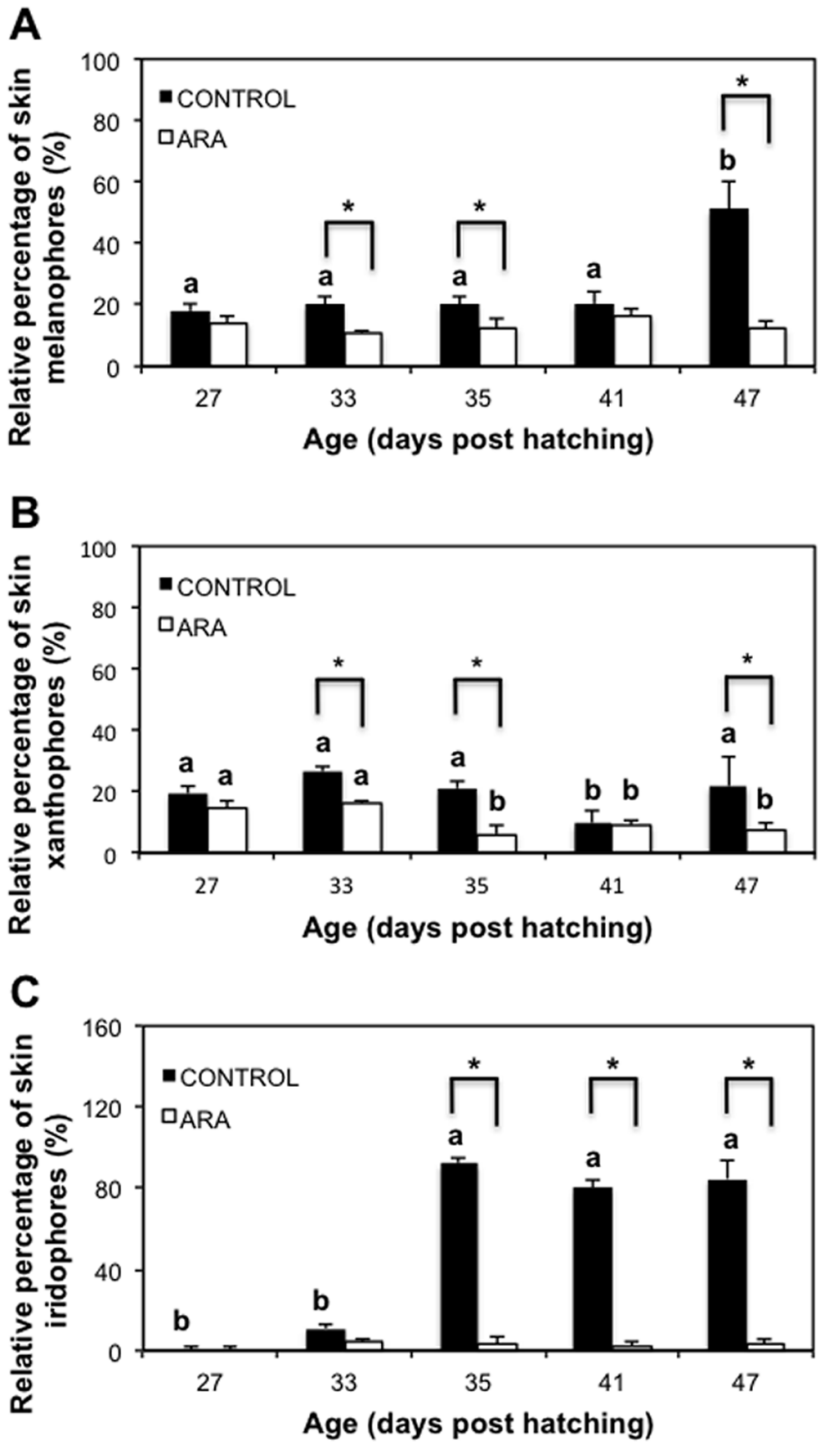
Relative percentage of skin melanophores (A), xanthophores (B) and iridophores (C) during post-metamorphosis of pigmented (control) and pseudo-albino (ARA) individuals of Senegalese sole. Values are expressed as mean ± SD (N = 4). Superscript letters denote significant differences in the relative amount of chromatophores with age for a given larval group and asterisks indicate significant differences in the amount of chromatophores between pigmented and pseudo-albino specimens for a given larval age (Two-way ANOVA, *P*<0.05; diet x age interaction *P*<0.006).

**Figure 3:**
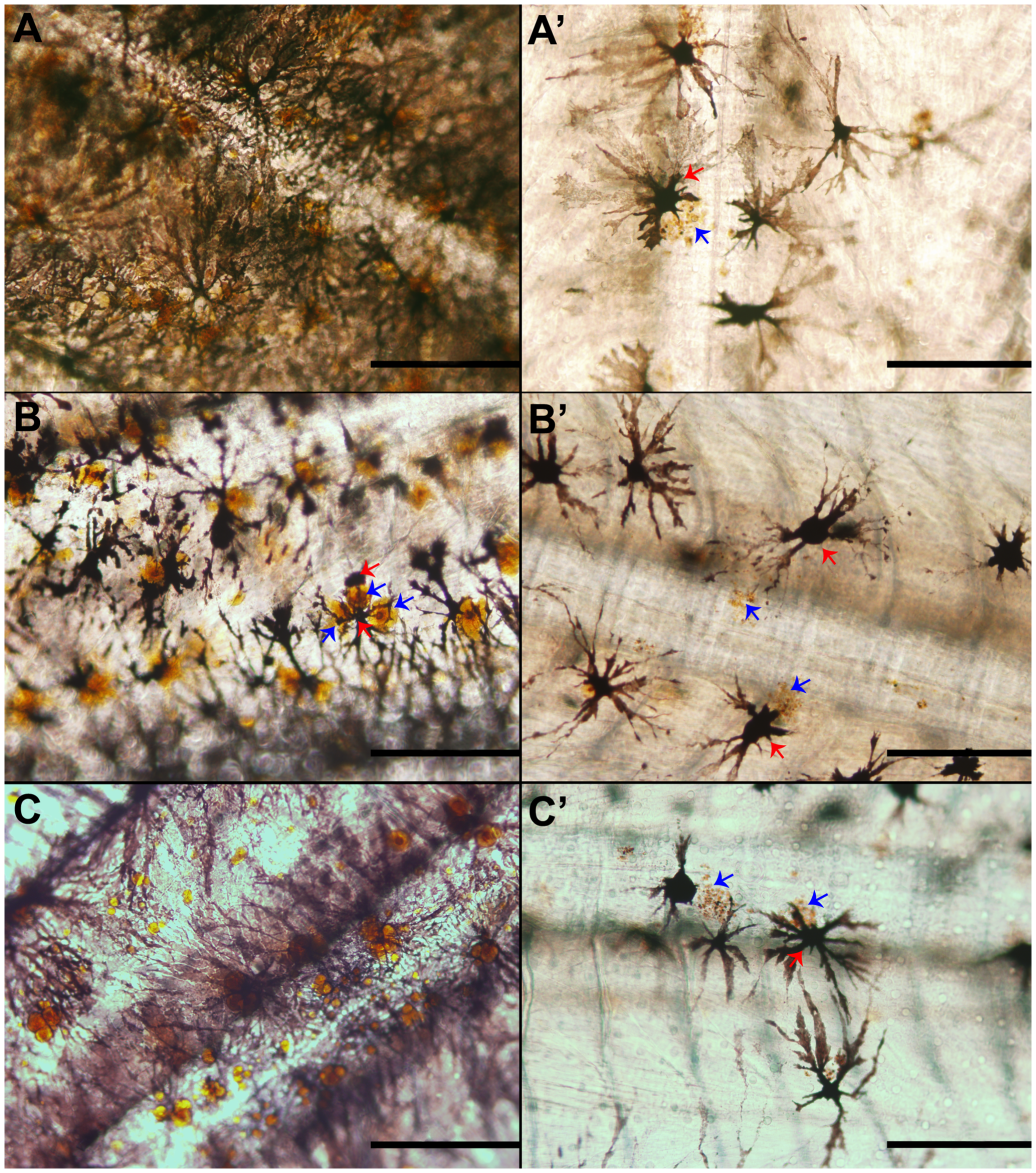
Microscopic images of the trunk skin of pigmented (A–C) and pseudo-albino (A’–C’) Senegalese sole specimens. A–C) skin showing abundant dendritic melanophores surrounded by several xanthophores. A’–C’) skin showing few melanophores and disintegrating xanthophores. A) 33 dph, B) 35 dph, C) 41 dph. Red arrows, melanophores; blue arrows, xanthophores. Scale bar, 500 µm.

**Figure 4:**
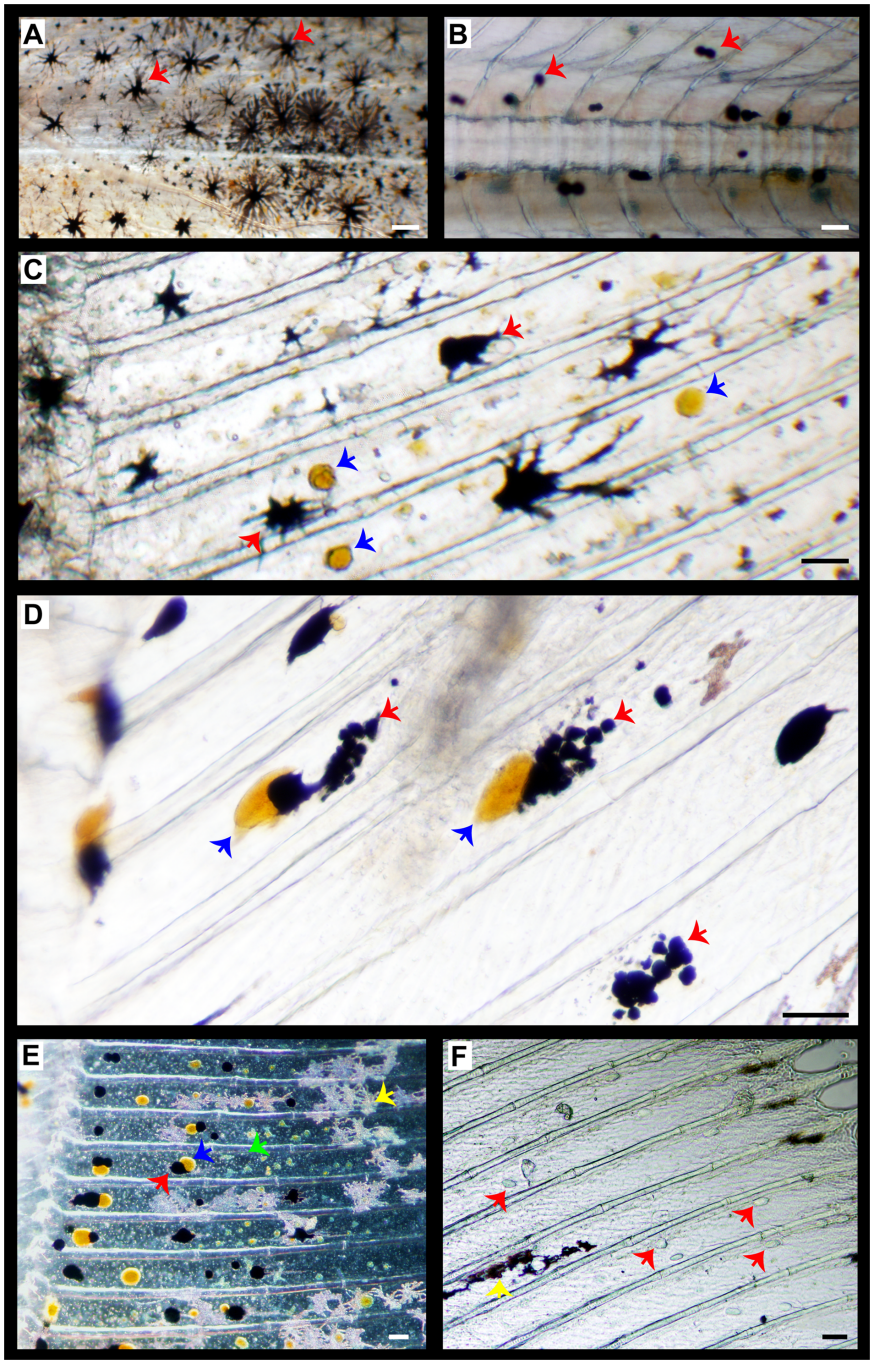
Microscopic images showing details of chromatophores distribution in the skin of pigmented larvae and pseudo-albinos in the ocular side of the fish. A–B) trunk skin at the level of the vertebral column in 47 day-old pigmented (A) and pseudo-albino (B) specimens. Melanophores are dendritic in pigmented individuals while the ability to disperse melanosomes is lost in pseudo-albinos. C–D) dorsal fin skin in 60 day-old pigmented (C) and pseudo-albino (D) specimens. Note the different shapes and sizes of xanthophores and melanophores between both groups: xanthophores are round in pigmented individuals whereas quite deformed and bigger in pseudo-albinos; melanophores are dendritic in pigmented individual while the round-shaped in pseudo-albinos. There are differences in the distance among xanthophores and melanophores between both groups. The interaction between these chromatophores was closer in pseudo-albinos and melanophores showed signs of disintegration. E) detail of the dorsal fin skin of a 33 day-old future pseudo-albino showing melanophores (black dendritic cells), xanthophores (yellow round cells), leucophores (dendritic cells that appear pale pink under reflecting light) and iridophores (iridescent round cells). Round-shaped melanophores and xanthophores were often associated in pairs, this being associated to the observed phenomenon of disintegration of one of the two chromatophores. F) detail of the dorsal fin skin of a 60 day-old pseudo-albino showing only few leucophores. Some non-identified transparent round structures were appreciated (red arrows). Red arrows, melanophores; blue arrows, xanthophores; green arrows, iridophores; yellow arrows, leucophores. Scale bars: A–B, 200 µm; C–F, 100 µm.

In brief and in terms of skin pigmentation, the progress of metamorphosis of Senegalese sole larvae occurred within the following periods: pre-metamorphosis (until 11 dph), pro-metamorphosis (from 11 to 19 dph) and post-metamorphosis (from 19 to 47 dph). During pre- and pro- metamorphosis, larvae from both dietary groups underwent a normal development of skin pigmentation. At 22 dph, when larvae already had flat symmetry, the pigmentation pattern of individuals from both dietary groups was similar, showing a very dense net of melanophores, xanthophores and leucophores that covered the head, the digestive cavity and the intermediate region of the trunk. They also presented two patches of chromatophores on the dorsal fin and another one on the anal fin (Figs. 1A–A’). At 27 dph, although the relative amounts of melanophores, xanthophores and iridophores were similar in both groups (Figs. 1B–B’, 2A–C), the skin of larvae from the ARA group appeared less pigmented than that of the larvae from the control group due to the progressive disappearance of leucophores that conferred the greenish aspect to the skin (Fig. 1B’). Differences in the amount and shape of melanophores and xanthophores between larvae from both experimental groups were in evidence from 33 dph (Figs. 1C–C’, 2A, 3A). The skin of the trunk presented abundant melanophores that were closely associated to 2 to 5 xanthophores in pigmented larvae (Fig. 3A–C, Table 2), whereas fewer xanthophores were related to one melanophores in pseudo-albinos (Table 2), most of them showing signs of disintegration (Fig. 3A’–C’; Table 3). Moreover, there were differences in the distance among xanthophores and melanophores between both groups. Generally, xanthophores were in direct contact with melanophores in pseudo-albinos, whereas they were closely associated, but not in contact, in pigmented specimens (Fig. 4A, B). Patches composed of melanophores, xanthophores and leucophores observed in pigmented individuals were not found in future pseudo-albinos (Fig. 1C’). However, iridophores were present in the pelvic fins of specimens from both groups (Figs. 1C–C’). At the level of the fins, future pseudo-albinos showed leucophores that appeared pale pink by reflected light, few iridescent iridophores, and round-shaped melanophores and xanthophores that were often associated in pairs and showed signs of degradation later in development (Figs. 3A’–C’, 4D, E). These chromatophores were progressively disappearing with age and, at 60 dph, the skin of the fin contained only a few leucophores located in the most apical region of the fins (Fig. 4F). At 35 dph the pseudo-albino phenotype was already established (Fig. 1D’). The differences in the amounts of xanthophores and iridophores between pigmented and pseudo-albino specimens became more evident (Figs. 1D–D’, 2B–C) and the grouping of iridophores in the fins of pigmented individuals was not found in pseudo-albinos (Fig. 1D’). From 35 to 47 dph, the amount of chromatophores in pseudo-albinos remained invariable (Figs. 1E’–F’, 2A–C). However, because there was an increase in the amount of melanophores and xanthophores in pigmented individuals from 41 to 47 dph, there was a significant difference in the amount of all chromatophores between pigmented larvae and pseudo-albinos at 47 dph (Fig. 2A–C). At the level of the trunk, melanophores were dendritic in pigmented individuals while the ability to disperse melanosomes seemed to be reduced in most pseudo-albinos (Figs. 4A, B, 6). In the skin of the fins, xanthophores and melanophores differed in shape between both groups: xanthophores were round in pigmented individuals whereas quite deformed in pseudo-albinos; melanophores were dendritic in pigmented individuals while round-shaped in pseudo-albinos (Fig. 4C, D).

**Table 2:** Number of xanthophores associated to one melanophore in specimens of Senegalese sole from the control and ARA groups during the post-metamorphosis period.

	Xanthophores/Melanophores	
Age (dph)	CONTROL	ARA
27	1,36±0,16^c^	1,69±0,13^a^
33^*^	3,54±0,82^ab^	0,40±0,18^b^
35^*^	1,81±0,16^bc^	0,27±0,09^b^
41^*^	4,15±0,64^a^	0,45±0,14^b^

Values are expressed in means ± SEM (N = 4). Superscript letters denote significant differences in the number of xanthophores related to one melanophore between developmental ages in each group (One-way ANOVA, *P*<0.05) and asterisks denote significant differences between groups of the same age (*t*-test, *P*<0.05).

**Table 3:** Percentage of disintegrating xanthophores in Senegalese sole specimens from the control and ARA groups during the post-metamorphosis period (N = 4).

	% Disintegrating xanthophores	
Age (dph)	CONTROL	ARA
27	25	25
33	25	100
35	25	50
41	0	57

### Skin Chromatophores

Figure 2 shows the relative amount of chromatophores in larvae from control and ARA treatments during post-metamorphosis (from 27 to 47 dph). Specimens from the control group showed 19.75±1.13% of melanophores from 27 to 41 dph. Then, the relative amount of melanophores increased up to 51% from 41 to 47 dph (Fig. 2A). Larvae from the ARA group presented 13.26±2.15% of melanophores during the whole post-metamorphosis period (Fig. 2A). The relative amount of melanophores was significantly lower in larvae from the ARA group compared to those from the control at 33, 35 and 47 dph (Fig. 2A). The relative amount of xanthophores in control specimens remained constant from 27 to 35 dph (22.28±3.62%). From 35 to 41 dph, the relative amount of xanthophores decreased to 9% and increased again up to 22% at 47 dph (Fig. 2B). Among larval chromatophores from the ARA group from 27 to 33 dph, 15.22±0.82% were xanthophores. From 33 to 35 dph, the amount of these pigment cells decreased by a half and remained invariable until 47 dph (Fig. 2B). The amount of xanthophores in pseudo-albinos was significantly lower than in pigmented individuals at 33, 35 and 47 dph (Fig. 2B). The amount of iridophores in pigmented individuals was low from 27 to 33 dph and then it increased 8 times from 33 to 35 dph and remained invariable from that day onwards (85.54±6.14%) (Fig. 2C). In pseudo-albinos, the amount of iridophores was low and stable during the entire period analyzed (2.63±1.64%) (Fig. 2C). The amount of iridophores was significantly lower in pseudo-albinos than in pigmented individuals from 35 dph onwards (Fig. 2C).

**Figure 5:**
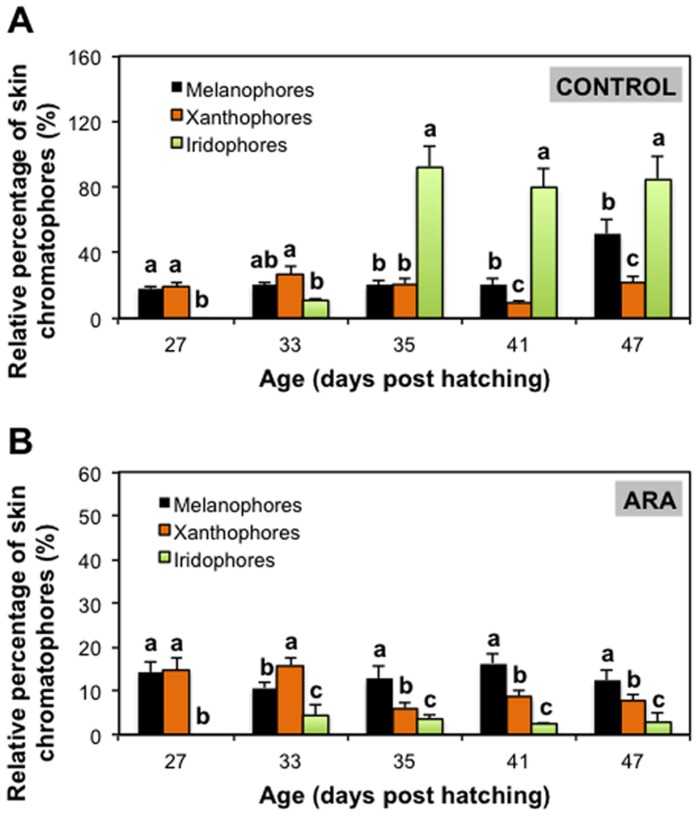
Relative percentage of skin chromatophores in pigmented (A) and pseudo-albino (B) specimens of Senegalese sole during post-metamorphosis. Values are expressed as mean ± SD (N = 4). Superscript letters denote significant differences in the relative amount of chromatophores for a given larval age (One-way ANOVA, *P*<0.05). Note that differences in the relative proportion of the three chromatophores between control and ARA groups were evident from 35 dph onwards and that they became invariable from 41 dph, indicating the establishment of the final skin color phenotype on the ocular side.

Figure 5 shows the relative percentage of skin chromatophores in specimens from control (Fig. 5A) and ARA (Fig. 5B) groups at each sampling day. At 27 dph, larvae from both populations presented similar relative amounts of skin melanophores and xanthophores, and a lower amount of iridophores. At 33 dph, xanthophores were the most abundant pigment cell in ARA group, followed by melanophores and, finally, iridophores. At 35 dph, the amount of iridophores was higher than that of melanophores and xanthophores in pigmented specimes, whereas melanophores were more abundant than xanthophores and iridophores in pseudo-albinos. At 41 dph, iridophores were more abundant than melanophores, followed by xanthophores in pigmented individuals, while melanophores were more abundant than xanthophores, followed by iridophores in pseudo-albinos. These profiles of chromatophore proportions were maintained at 47 dph.

The number of xanthophores associated to one melanophore in each group of larvae during post-metamorphosis of Senegalese sole is shown in Table 2. Larvae from the control group showed a X/M (xanthophore/melanophore) ratio close to 1 at 27 dph. The number of xanthophores related to one melanophore increased at 33 dph, and decreased until 2 from 33 to 35 dph. At 41 dph, the X/M ratio increased again displaying an average of 4. The X/M ratio in the skin of larvae from the ARA group was close to 1 at 27 dph and it subsequently decreased to almost 0 until the end of the analyzed period.

**Figure 6:**
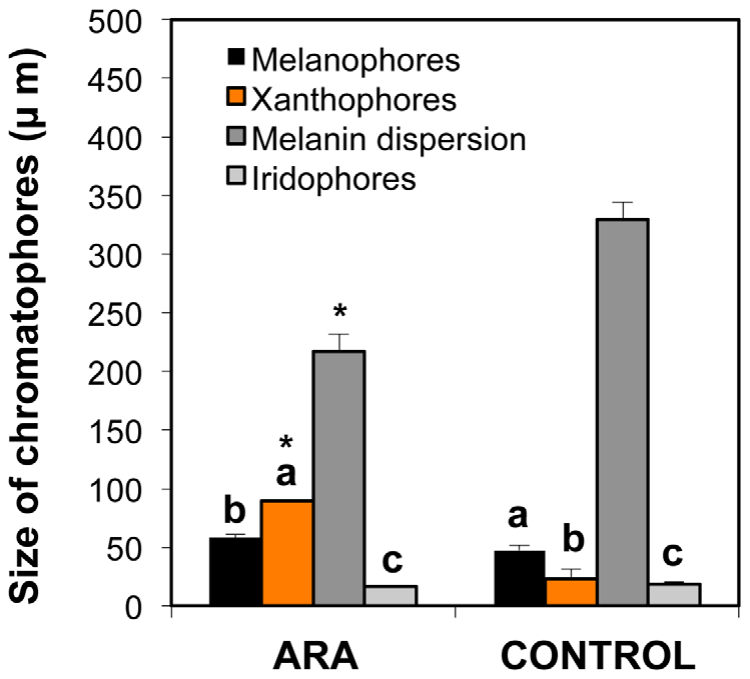
Size of skin melanophores, xanthophores and iridophores, and melanin dispersion distance in 60 day-old pigmented (control) and pseudo-albino (ARA) specimens of Senegalese sole. Values are expressed as mean ± SD (N = 4). Superscript letters denote significant differences in size between chromatophores of a given larval group (One-way ANOVA, *P*<0.05) and asterisks indicate significant differences in size of chromatophores and melanin dispersion distance between pigmented and pseudo-albino specimens (Student’s *t*-test, N = 4, *P*<0.001).

The size of skin chromatophores in pseudo-albinos and pigmented specimens at post-metamorphosis is shown in Figure 6. The size of melanophores was around 50 µm in pseudo-albinos and pigmented specimens. However, their capacity to disperse melanine was significantly reduced in pseudo-albinos (Figs. 4A, 4B, 6). The size of xanthophores was higher in pseudo-albinos than in pigmented specimens (90 versus 22 µm in average). Iridophores were the smallest pigment cells and their size was similar in both larval groups (17 µm in average, Fig. 6).

From individuals in the control group 25% showed disintegrating xanthophores from 27 to 35 dph, whereas no evidences for xanthophores disintegration were observed at 41 dph (Table 3). From the ARA group 25% of larvae presented disintegrating xanthophores at 27 dph, a 100% at 33 dph, and around a 50% at 35 and 41 dph (Table 3).

### Modulation of Gene Expression in Pseudo-albino Specimens

Partial coding sequences from 10 pigmentation-associated genes from Senegalese sole (Table 4) were obtained using consensus primers: melanocyte-stimulating hormone 1 receptor (*mc1r*), agouti signaling protein (*asip*), paired box protein 3 (*pax3*), microphtalmia-associated transcription factor (*mitf*), tyrosinase (*tyr*), tyrosinase-related protein 1 (*trp1*), mast/stem cell growth factor receptor Kit (*cKit*), sodium/potassium/calcium exchanger 5 (*slc24a5*), enzyme caspase 3 (*casp3*) and heat shock 70 kDa protein (*hsp70*). Fragments ranged in size from 272 to 1138 base pairs. Additionally, somatolactin (*sl*) was downloaded from GenBank.

**Table 4:** Accession numbers, amplicon size, primers and hydrolysis probes used in qPCR analyses.

Gene name	GenBank accession no.	Amplicon size	Hydrolysis probes	5′ to 3′ sequence
*Ubq*	**AB291588**	86	Forward	GCCCAGAAATATAACTGCGACAAG
			Reverse	TGACAGCACGTGGATGCA
			FAM probe	ACTTGCGGCATATCAT
*Tyr*	**JF693907**	73	Forward	CGTACGCACAGATGGAAAACG
			Reverse	CACGTAGTAATGCATCCACACAAAA
			FAM probe	ACATCGGCGAATATC
*Trp1*	**GU329041**	63	Forward	CGTGTGCAACAATACAGAAACAAGT
			Reverse	ATGGGTCGTGCCACGTT
			FAM probe	CCTGCCGGGTTCCTT
*Mitf*	**GU329042**	75	Forward	CGATGACATCATAAGTCTTGAATCCAGTTT
			Reverse	CGTGCTGGGCAACTGAAGA
			FAM probe	CCGGAGTCAATCAACG
*cKit*	**HM100237**	69	Forward	GTGAAGAGAGTGAGATGTTTGACGA
			Reverse	CACTTTGGTAGGAGAAGCTCAGAA
			FAM probe	CTCGTCACCGAAGATC
*Mc1r*	**GU329043**	76	Forward	CGCCGTCGCCATCATC
			Reverse	GCGTTGTCCGTGTGGTAGA
			FAM probe	ACCTCCAGCATCCTCT
*Scl24a5*	**GU329046**	66	Forward	GACGCAGCCTCTGATCGA
			Reverse	CCGTCCTGGAGCGAACC
			FAM probe	CCAGTCTGCGAAACAT
*Casp3*	**GU329040**	77	Forward	CGACAGTGTAGATGACCAAACGT
			Reverse	GGAGCAGTGGAATAAGCATAAAGGA
			FAM probe	CCTCCACAGGAATCC
*Pax3*	**HM100238**	68	Forward	GCATCATGCGCTCCAAGTTC
			Reverse	CCCTCTTCACCATTTCATCATCCT
			FAM probe	CATCGTCACCAACTCC
*Sl*	**U06753**	75	Forward	TTCCCACTGCGGCTTCA
			Reverse	GGTAAGGCCTTGGTGATGCA
			FAM probe	CCGACCGTGTTTCTC
*Asip*	**HE598753**	81	Forward	GCTGTGACATCTGTGCCTTCT
			Reverse	CCATTCGACAGAAACACACAGTTC
			FAM probe	CCAGTGTCGCCTCTTC
*Hsp70*	**GU329044**	73	Forward	TGGAGTCGTATGCTTTCAACATGA
			Reverse	TGCTTGTCGTCGTCACTGAT
			FAM probe	CTTGCCAGCCAGTTTC

**Figure 7:**
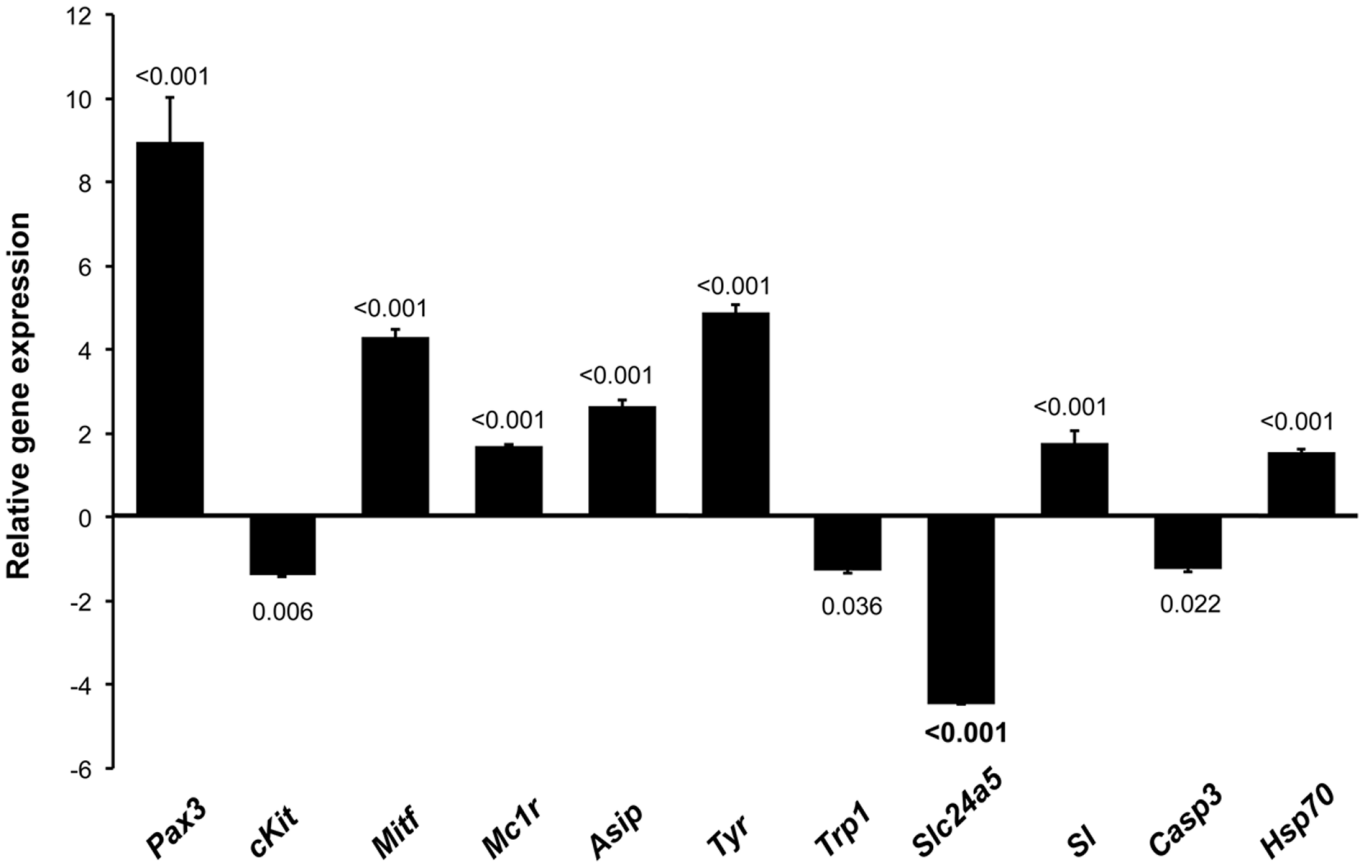
Fold change relative gene expression of pigmentation-related genes in 60 day-old pseudo-albino (ARA) specimens of Senegalese sole compared to pigmented specimens. Melanocyte-stimulating hormone 1 receptor (*mc1r*), agouti signaling protein (*asip1*), paired box protein 3 (*pax3*), microphtalmia-associated transcription factor (*mitf*), tyrosinase (*tyr*), tyrosinase-related protein 1 (*trp1*), mast/stem cell growth factor receptor Kit (*cKit*), sodium/potassium/calcium exchanger 5 (*slc24a5*), enzyme caspase 3 (*casp3*), heat shock 70 kDa protein (*hsp70*) and somatolactin (*sl*). Student’s *t*-test, N = 4, *P*-values shown in figure.

The average efficiency of amplification for all assays was 99%, the slopes of the standard curves being 3.3, 3.1, 3.5, 3.2, 3.2, 3.5, 3.3, 3.5, 3.5, 3.4 and 3.3 for *pax3*, *tyr*, *mitf*, *mcr1*, *asip, hsp70*, *sl*, *casp3*, *trp1*, *slc25a5* and *cKit,* respectively. Results of gene expression in 60 day-old pseudo-albino specimens showed a positive and statistically significant up-regulation of *pax3*, *tyr*, *mitf*, *mcr1*, *asip, hsp70* and *sl* (9, 5, 4, 1.7, 2.6, 1.5 and 1.8 fold changes, respectively) and a down-regulation of *casp3*, *trp1*, *slc24a5* and *cKit* (1.2, 1.3, 4.4 and 1.4 fold changes, respectively) compared to the pigmented individuals (Fig. 7).

## Discussion

### Morphological Development of Pseudo-albinos

Morphological data revealed that ARA did not affect larval pigmentation at the pre-metamorphic stage, but prevented chromatophore terminal differentiation at metamorphosis, leading to the appearance of pseudo-albinism. The amount of melanophores and iridophores in pseudo-albinos remained invariable during the entire studied period. However, normally pigmented specimens showed an increase in the population of melanophores and iridophores at post-metamorphosis (47 and 35 dph, respectively). This indicates that the new population of chromatophores that should appear after metamorphosis was not formed (or cells were not pigmented) in pseudo-albinos. While molecular signaling towards the differentiation of new populations of melanophores, xanthophores and iridophores occurs during pro-metamorphosis (11–19 dph), morphological changes occur later at post-metamorphosis [15]. Considering that the amount and proportions of these chromatophores were similar in pigmented and pseudo-albino specimens until 27 dph, and that their number remained invariable from that day onwards, it seemed that pigment cell precursors were likely influenced by the asymmetric signaling during pro-metamorphosis rather than in mature larval chromatophores [13]. The decrease in xanthophores from 33 to 35 dph observed in pseudo-albinos was likely the result of the degradation of already existent xanthophores. Interestingly, and contrary to pigmented specimens, round-shaped melanophores and xanthophores were often coupled in pseudo-albinos and their closer association, together with the increased size of xanthophores, suggested some type of mechanism may be in operation that leads to the collapse of these chromatophores. The different allocation, shape, size and distance between melanophores and xanthophores observed in pseudo-albinos suggested the possible existence of an altered communication between these pigment cells. Interactions between these pigment cells have already been reported and it is believed that xanthophores regulate melanophore pattern [22]. In fact, this relationship is required to form the Turing pattern of zebrafish [23] and has also been suggested for the pigmentation patterning of Senegalese sole [15]. Moreover, it has been shown that when melanophores and xanthophores are adjacent, these cells exclude each other [23]. These interactions seem to be necessary for the normal development of pigmentation [15], as 25% of the population of pigmented individuals showed disintegrating xanthophores in their skin from 27 to 35 dph. However, at 41 dph, disintegrating xanthophores were not observed in pigmented specimens, probably indicating the end of the action of melanophores over xanthophores for the establishment of the adult pigment pattern. In a previous study we observed that the normal pattern of interaction between xanthophores and melanophores towards the achievement of the adult pigmentation showed a fluctuating X/M ratio from 27 to 47 dph [15]. Present results suggest that the X/M ratio and cell proximity play a key role in the correct dorsal-ventral pigment patterning. Although the amount was not significant compared to that of xanthophores, disintegrating melanophores were observed from 41 to 60 dph (Fig. 4D). In line with this, xanthophores of zebrafish were able to reduce the surrounding melanophores within a short-range, whereas xanthophores that were distally located from melanophores were able to enhance the development and survival of the latter cells [23]. This seemed to also be the case for pigmented Senegalese sole specimens in which small xanthophores could be seen surrounding the well-developed melanophores (Fig. 4C). Altogether these results show that chromatophores develop and interact in a very delicate equilibrium where xanthophores play a central role in pigment patterning, the relative proportion and behavior (allocation, size, shape) of this pigment cell being critical for the correct ontogeny of pigmentation.

Pseudo-albinos had lost most of their leucophores, with those few remaining being located almost exclusively in the distal part of the fins. Moreover, when analyzing different specimens displaying different degrees of pseudo-albinism, it was noticed that, as the fins grew, tissue lacking pigmented chromatophores appeared basally. This could be an indication of new tissue devoid of chromatophores (or cells devoid of pigment) being produced basally and that the early leucophores located basally disappeared (Fig. 4F) [13].

### Modulation of Gene Expression in Pseudo-albino Specimens

The morphological changes in skin pigmentation observed in pseudo-albinos were reflected in their molecular signaling response as seen via analysis of the eleven pigmentation-related genes in which different expression patterns were evident in pseudo-albinos as compared to pigmented specimens. These differences likely prevented the development of post-metamorphic melanophores, xanthophores and iridophores.

**Figure 8:**
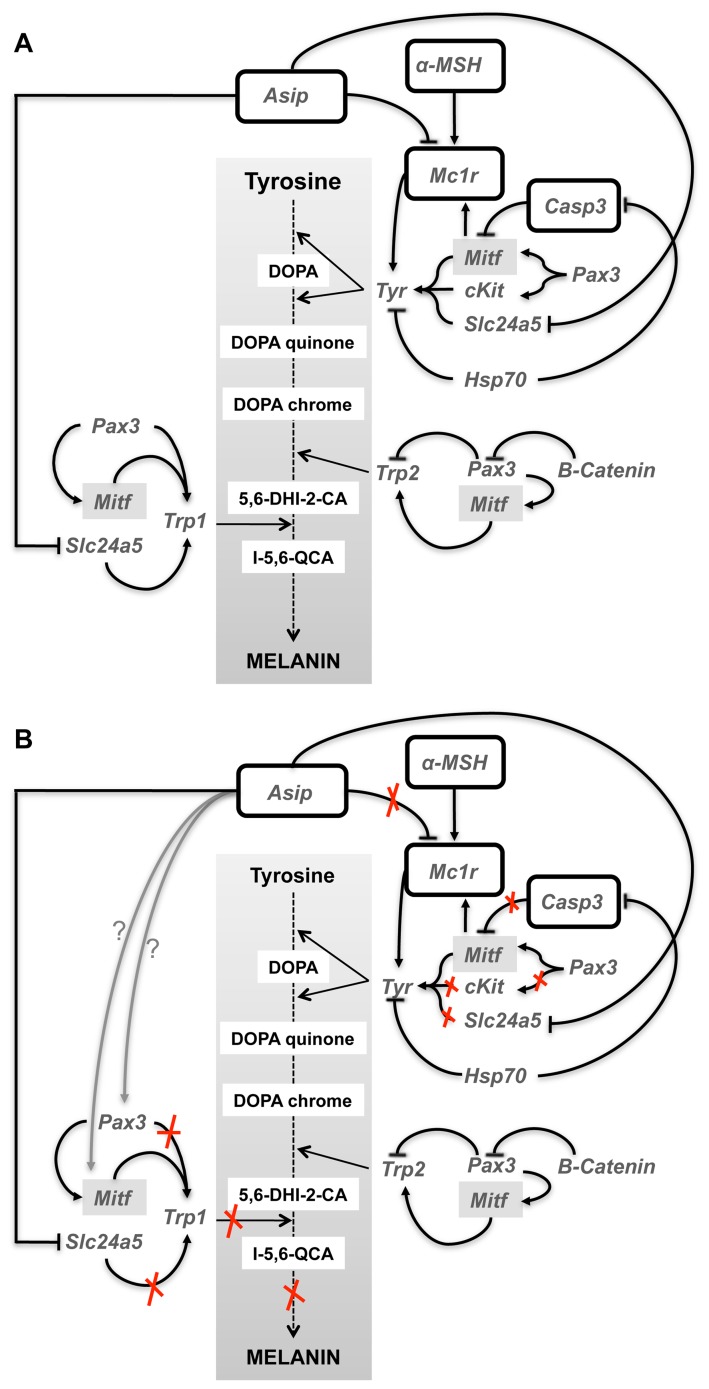
Schematic picture showing the known action of the analyzed genes in melanocyte differentiation and melanogenesis in vertebrate melanocytes (A) and the suggested pathways of melanophore differentiation and melanogenesis that were altered in pseudo-albinos of Senegalese sole (B). The explanation of the figure is given in the main text.

Regarding melanophores, two mechanisms (probably related) accounted for the absence of melanin. The first one is that initial but not terminal differentiation of melanophores was promoted and the second one is that melanin synthesis was disrupted at the last step of this process (Figs. 7, 8). It has been recently shown that the gene *sl* enhances the differentiation of melanophores in medaka, although the specific molecular action is not yet known [24]. The up-regulation of *sl* expression in Senegalese sole pseudo-albinos pointed to melanophore differentiation being stimulated. This is further supported by the significant up-regulation of *pax3,* which is one of the first genes expressed in melanoblast/melanophore precursors [25], [26] and plays a key role in controlling the development of melanocytes [27]. In this capacity, *pax3* maintains the equilibrium between melanocyte differentiation and melanin synthesis. During stem cell differentiation into melanocytes, *pax3* simultaneously induces a melanogenic cascade, through the activation of *trp1* expression, while acting downstream to prevent terminal differentiation [28]. Such a mechanism guarantees cellular proliferation, thus sustaining this cell population [27]. *Pax3* is able to inhibit the expression of *trp2*, responsible for the conversion of L-dopachrome into 5,6-dihydroxyindole-2-carboxylate. This ensures high levels of *mitf* expression to induce melanocyte differentiation [29], [30] but at the same time prevents terminal differentiation. In the presence of β-catenin, the action *of pax3* to block *trp2* transcription is missing [31] and then *trp2* contributes to melanocyte differentiation (Fig. 8). In Senegalese sole pseudo-albinos, *pax3* seemed to promote melanophore differentiation through the up-regulation of *mitf* while preventing its terminal differentiation through the down-regulation of *trp1* expression (Fig. 8). As occurs with *mitf, pax3* also promotes and inhibits melanogenesis through transcriptional regulation of *cKit* [30]. *CKit* plays a critical role in melanocyte physiology by influencing melanogenesis, migration and survival of these cells [32] and it is primarily expressed in the melanized melanophores [13]. In Senegalese sole developing larvae, the profile of *cKit/pax3* and *mitf*/*pax3* ratios reflected the pattern of melanophore ontogeny [15]. Larvae underwent a process of melanogenesis during pre-metamorphosis and a stabilization of melanophore differentiation and melanogenesis processes during pro-metamorphosis. Once metamorphosis was finished, these ratios increased again indicating the prevalence of melanogenesis over melanophore differentiation. The gene expression profile of *pax3*, *mitf* and *cKit* in metamorphosed pseudo-albinos compared to pigmented individuals indicated that melanophore differentiation prevailed over melanin synthesis, which is surprisingly opposite to what was expected to occur at that period. This suggests that the switch of the molecular events that should take place from pro- to post-metamorphosis to develop the adult pigmentation pattern was disrupted in pseudo-albinos.

*Mitf* is also a master regulator of melanogenesis due to its ability to activate many melanocyte-specific genes, such as *tyr*, *trp1* and *trp2* [33], [34], and *mc1r* [35]. The melanocyte-stimulating hormone alpha (*α-MSH*) induces the proliferation of melanophores through binding to its receptor, *MC1R* [3], and activates melanogenesis through the positive regulation of tyr activity (Fig. 8). The end of pigmentation metamorphosis in pigmented Senegalese sole specimens was marked by a decrease in *mc1r* expression [15]. However, pseudo-albinos at post-metamorphosis showed an up-regulation of *mc1r* providing more evidence for altered molecular signatures preventing terminal development of skin pigmentation. The elevated amount of *mitf* transcripts in pseudo-albinos could be responsible for the up-regulation of *mc1r*. As a consequence of this, and as happens in pigmented individuals, an up-regulation of *tyr* induced by *mc1r* could be expected [36]. It has been shown that *tyr* gene transcription is also induced by the transient activation of the cKIT receptor by its ligand, stem cell factor, in differentiated melanocytes [37]. Down-regulation of *cKit* observed in pseudo-albinos compared to pigmented specimens likely indicated that *cKit* was not the primary cause for the induction of *tyr* expression. The lower levels of expression of *cKit* observed in pseudo-albinos could reflect the lower amount of differentiated melanophores compared to the pigmented individuals. But it also corroborates that *cKit* was not expressed in what seem to be unmelanized melanophores. Further, *tyr* expression could also be directly induced by *pax3*. The significant up-regulation of *pax3* expression in pseudo-albinos, together with *mitf* and *tyr,* illustrates the above-mentioned role of *pax3* in controlling melanophore differentiation. However, the pseudo-albino phenotype was characterized by the presence of few melanized melanophores. This is in line with the thought that *pax3* in mammals is expressed in early development, but inhibited in adult melanocytes [31]. Therefore, although molecular signaling suggests that melanophore differentiation was favored, melanogenesis was not.

*Asip1* is thought to be involved in the establishment of the dorsal-ventral pigment pattern by directing chromatophore differentiation, causing production of iridophores and inhibiting the production of melanophores [12]. However, the development of iridophores, which typically occurs at post-metamorphosis [15], was distorted in Senegalese sole pseudo-albinos. Our results suggest that ARA affected normal gene expression in a way which blocked the pigment patterning that should have been established on the ocular side of the fish at metamorphosis via *asip1* gene expression; then, the excessive *asip1* expression levels could alter normal chromatoblast differentiation leading to an inhibition of differentiation of new post-metamorphic iridophores. This hypothesis is supported by recent findings on *asip1* regulation in adult Senegalese sole pseudo-albinos that showed a higher expression of this gene within unpigmented patches of the dorsal skin than in pigmented regions, but similar to the levels found in the blind side [14]. In developing Senegalese sole larvae, *asip1* seemed to be responsible for the inhibition of melanophore differentiation on the blind side concomitant with the induction of iridophore differentiation on the ocular side before the differentiation of iridophores on the blind side [15]. We propose that the disruption of the dorsal-ventral pigment patterning during metamorphosis entailed the establishment of the mechanisms for blind side pigment pattern on the ocular side but blocked at the point of inhibition of melanophore differentiation and before the differentiation of iridophores. It is interesting to note that iridophores were present in pelvic fins of pseudo-albinos, suggesting that the molecular signaling towards their differentiation took place before mouth opening, as is the case of the eyes [15], or, alternatively, it is independent from the rest of the body. Indeed, little information exists on the molecular mechanisms driving iridophore development. It has been recently demonstrated that *mitf,* the key regulator of melanophore development and melanogenesis [25], [38], also regulates cell fate plasticity in zebrafish, including the formation of iridophores [33]. Minchin and Hughes [26] demonstrated that *mitf* inhibits the iridophore fate in zebrafish. In Senegalese sole pigmented individuals, the expression of *mitf* decreased during development to promote the bi-potent pigment cells precursors to differentiate into iridophores instead of melanophores [15]. However, this gene was up-regulated in pseudo-albinos, giving more evidence for the disruption of the normal development of iridophores in these specimens. Considering this, it is tempting to speculate that the overexpression of *mitf* in Senegalese sole pseudo-albinos could be blocking the differentiation of precursor cells into iridophores, as melanophore and iridophores have a common precursor and both cell types were not developed in pseudo-albinos. This is suggestive that an excess of *asip1 and mitf* expression is preventing the development of melanophores and iridophores by exerting their inhibitory function at the level of their cellular precursors. Whether *asip1* is able to modulate the expression of *mitf* and/or vice versa needs to be explored. These findings are in line with the evolvement of chromatophore development in Senegalese sole peudo-albinos at post-metamorphosis, where the amount of melanophores was invariable, the amount of xanthophores was reduced and iridophores were not differentiated. The putative implication of *mitf* in xanthophore disintegration deserves to be investigated. Taking into account these findings, Senegalese sole pseudo-albinos seemed to face a problem of post-metamorphic iridophore and xanthophore development and terminal differentiation of melanophores. Whether the above described molecular signaling contributing to melanophore differentiation is due to signals from the surrounding immature melanophores needs to be further investigated.

Although *tyr* expression was highly induced in pseudo-albinos, the completion of this process was inhibited at the last step of melanin production through the down-regulation of *trp1*. Moreover, the low expression level of *trp1* would be induced by the down-regulation of *slc24a5* (Fig. 8). In fact, it has been demonstrated that the knockdown of *slc24a5* provoked dramatic effects on pigmentation in re-differentiating human epidermal melanocytes causing hypomelanosis [39]. *Slc24a5* is a putative cation exchanger known to increase uptake and accumulation of calcium in melanocytes, necessary for melanin synthesis [40], [41] where calcium plays an important role as a second messenger in the initiation of melanogenesis by stimulating the synthesis of L-tyrosine [42]. The significant down-regulation of *slc24a5* expression in pseudo-albinos suggests that tyrosine production may be altered in these specimens. Interestingly, expression of *slc24a5* has been found to be negatively regulated by *asip1* [43] (Fig. 8). The reason why and how this gene is down-regulated by ARA to generate pseudo-albinos needs further investigation. Therefore, in addition to the known role of the gene *asip1* in iridophore differentiation in fish [12], our results show that *asip1* also regulates melanophore differentiation and melanogenesis [44]. This has been already demonstrated in mammals, where *asip1* regulates the relative proportions of eumelanin (black-brown pigment) and phaeomelanin (yellow-red pigment) by antagonizing the action of *α*-MSH on its receptor MC1R [45]; additionally, *asip1* negatively regulates the expression of *mc1r* [46] and *slc24a5* [43]. Fish only produce eumelanin, and *mc1r* expression was up-regulated in pseudo-albinos, therefore the regulation of melanogenesis is likely blocked at some step downstream from *mc1r*. We propose that melanin synthesis is inhibited in newly differentiated melanophores by negatively regulating the expression of *slc24a5,* which is necessary for melanogenesis. Although low, *trp1* expression still existed in these specimens, likely reflecting melanogenesis within the few melanophores remaining in the skin. The primary function of *mc1r* in fish is to mediate distribution of melanin granules in melanophores, allowing the organism to adapt to the background [47]. The expression of this gene was up-regulated in pseudo-albinos, though the ability to disperse melanin within melanophores was reduced.

Gene expression data suggest that ARA changed the pigment pattern that should have been established in the ocular side of the fish at metamorphosis through its effects on *asip1, pax3* and/or *mitf*; thereafter, the excessive expression levels of these genes could alter their normal action on chromatoblast differentiation leading to an inhibition of terminal differentiation of new post-metamorphic melanophores, xanthophores and iridophores. Abnormal spatial distribution between xanthophores and melanophores was observed in pseudo-albinos. An excessive disintegration of xanthophores seemed to prevent the normal patterning of melanophores, suggesting that normal pigmentation at metamorphosis depends on some type of communication established between these pigment cells and their proportions. We propose that the unexpected behavior of these pigment cells in the skin of the ocular side of the fish is typically taking place in the blind side during metamorphosis for the development of the asymmetrical dorsal-ventral pigment patterning characteristic of flatfish. The molecular mechanisms underlying these processes need to be further explored. Taking into account the well-known role of *asip1* in the establishment of the dorsal-ventral pigment patterning in this and other species [12], [14], and the results obtained in this study, the role of *asip1* in xanthophore physiology deserves investigation. These findings show that the genetically determined pigmentation in larvae can be modulated by environmental factors (i.e., nutrition) and thereby affect changes in the normal molecular signatures occurring during the process of metamorphosis.

We conclude that differentiation of melanophores was promoted in pseudo-albinos through *pax3*, *mitf*, *mc1r* and *tyr* signaling with a simultaneous deficiency of terminal differentiation. *Asip1* and *mitf* seemed to play a key role in the prevention of differentiation of pigment cell precursors into melanophores and iridophores. *Slc24a5* seems to play a key role in the disruption of melanogenesis at the end of this process by decreasing *trp1* expression. We propose that the down-regulation of *slc24a5* could be induced by up-regulation of *asip1*, reaffirming the involvement of this gene in melanogenesis. The expression level of *pax3* appeared to be critical for the development of the new populations of melanophores, xanthophores and iridophores at post-metamorphosis. Among these chromatophores, xanthophores seemed to play a central role in pigment patterning, where interaction between pigment cells occurred in a delicate equilibrium. Our results demonstrated that the relative proportions between xanthophores and melanophores, cell proximity, size and shape are critical for the correct ontogeny of pigmentation. Further molecular research on chromatophore interactions would definitely shed light to the mechanisms governing cellular communication leading to the establishment of the pseudo-albino phenotype. Additional insights may also be gained by separation of blind side from ocular side skin for a more detailed look at chromatophore development.

## Methods

### Ethics Statement

This study was carried out in accordance with the recommendations in [48]. Animal experimental procedures were conducted in compliance with the experimental research protocol (reference number 4978-T9900002) approved by the Committee of Ethic and Animal Experimentation of the IRTA and the Departament de Medi Ambient i Habitatge (DMAH, Generalitat de Catalunya, Spain) in accordance with EU regulation (EC Directive 86/609/EEC).

### Animal Rearing and Sampling Procedures

Two-day-old Senegalese sole larvae were obtained from Stolt Sea Farm SA (Carnota, A Coruña, Spain), acclimated at IRTA-SCR facilities and reared at 16.7±0.4°C and 35 of salinity in 8, 60 l cylindrical white bottomed tanks (initial density: 110 larvae l^−1^) connected to a IRTAmar™ recirculation unit. Water was daily renewed (50%) with gentle aeration in each tank, pH and dissolved oxygen being 8.0±0.2 and 7.5±1.3 ppm, respectively. Photoperiod was 16L: 8D, and light intensity was 500 lx at the water surface. Larvae were fed twice a day, from 2 days post hatching (dph) to 10 dph, with enriched rotifers (*Brachionus plicatilis*), at a density of 10 rotifers ml^−1^ from 2 to 8 dph and of 5 rotifers ml^−1^ from 9 to 10 dph. Enriched *Artemia* metanauplii were supplied to larvae from 8 to 60 dph twice a day, at increasing density from 0.5 to 12 metanauplii ml^−1^ according to the increase of weight of the larvae and to the daily food ration [49]. From 20 dph onwards, when larvae were settled at the bottom of the tank, enriched *Artemia* metanauplii were supplied frozen [18].

In order to induce pseudo-albinism [17], larvae from four tanks were fed with rotifers and *Artemia* nauplii enriched with an experimental emulsion containing high levels of arachidonic acid (ARA). Briefly, the experimental emulsion was prepared from commercially available oils rich in DHA and ARA obtained respectively, from liver of *Gadus morhua* (Fluka©, Sigma-Aldrich, Chemie GmBH, Steinheim, Norway) and from fungus *Mortierella alpina* (Vevodar®, DSM Food Specialties, Netherlands). Olive oil was added to the oil mixture to dilute and adjust n−3 PUFA concentration in enriched live prey and α-tocopherol for preservation of the emulsion. The oil mixture was emulsified with soy lecithine and distilled water by homogenizing with an Ultra-turrax T25 at high speed for 60 s. Larvae from the other four tanks were fed rotifers and *Artemia* nauplii enriched with a commercial enrichment (AGM, Algamac 3050™, Aquafauna, Biomarine Inc., USA) and used as a control group to monitor the normal pigmentation development. The ingredients used in the formulation ARA emulsion and the fatty acid composition of AGM (control) and ARA enrichments are shown in Table 5.

**Table 5:** Total lipid and fatty acids contents (mg·g DW^−1^) and fatty acid composition (% of TFA) of the enriched rotifer (mean± SD; N = 5) and *Artemia* nauplii (mean ± SD; N = 3) with the two enriching compounds and in Senegalese sole larvae at 2, 15 and 50 dph fed the two dietary treatments (mean ± SD; N = 3).

	Rotifer		*Artemia*		Larvae(2 dph)	Larvae (15 dph)		Juvenile (50 dph)	
	CONTROL	ARA	CONTROL	ARA		CONTROL	ARA	CONTROL	ARA
Total lipid	140.6±18.5	143.2±25.2	164.9±14.37	164.9±15.1	84.4	109.9±19.4	82.8±11.8	86.7±13.9	100.6±21.9
Total FA	74.2±13.0	61.4±13.6	87.6±14.4	85.0±14.3	22.5	50.7±12.4	31.3±5.6	34.6±9.4	46.3±11.8
14∶0	3.3±1.2^a^	0.8±0.2^b^	0.6±0.2^a^	0.3±0.1^b^	0.5	0.7±0.2	0.4±0.1	0.7±0.1^a^	0.4±0.0^b^
16∶0	16.3±2.5^a^	11.8±0.9^b^	10.7±0.3^a^	9.1±0.6^b^	18.6	11.8±0.8	11.3±1.1	14.1±1.0	10.8±0.2
18∶0	1.7±0.3^b^	4.4±0.5^a^	6.2±0.9	5.9±0.7	10.3	7.5±0.4^ab^	9.6±0.4^a^	9.6±0.8	7.7±0.1
Total saturated	21.4±3.5^a^	17.2±1.6^b^	17.5±0.9^a^	15.3±1.2^b^	29.4	20.2±1.4	21.3±1.2	24.6±2.0	19.0±0.2
16∶1n-9	5.2±2.0	7.1±1.4	1.2±0.2^b^	1.8±0.2^a^	3.8	1.4±0.5	2.4±0.6	1.4±0.5	2.1±0.5
18∶1n-9	6.6±2.6^b^	25.6±4.0^a^	14.4±3.7^b^	23.1±2.0^a^	10.0	13.3±1.9	22.2±4.6	14.3±0.4^b^	22.3±0.9^a^
18∶1n-7	1.8±1.0	3.7±2.1	6.0±1.5	6.8±1.4	5.6	1.3±1.5	3.5±2.4	3.7±1.1	5.2±1.0
20∶1n-9	0.8±0.5^b^	5.3±0.5^a^	0.0±0.0^b^	1.9±0.8^a^	0.0	0.2±0.2	1.2±0.7	0.4±0.1b	1.4±0.3^a^
Total monounsaturated	14.4±4.6^b^	41.8±2.8^a^	21.7±5.5^b^	33.6±2.9^a^	19.4	16.2±0.6^b^	29.4±1.9^a^	19.8±2.0^b^	31.0±0.5^a^
18∶2n-6	2.4±0.6^b^	9.3±4.8^a^	4.6±1.2^b^	6.7±0.4^a^	3.2	3.1±0.4^b^	8.0±0.3^a^	3.6±0.3^b^	6.9±0.4^a^
18∶3n-6	0.6±0.1^b^	1.5±0.3^a^	1.3±0.3	1.3±0.1	0.7	0.9±0.0	1.1±0.1	1.0±0.1	1.2±0.1
20∶3n-6	0.9±0.3	1.4±0.7	0.2±0.2	0.9±1.0	0.0	0.2±0.2	0.6±0.4	0.4±0.1^b^	0.7±0.1^a^
**20∶4n-6**	**1.0±0.4^b^**	**10.2±1.2^a^**	**1.4±1.3^b^**	**7.1±4.1^a^**	**3.2**	**4.4±0.6^b^**	**10.9±0.8^a^**	**4.1±0.2^b^**	**10.2±0.3^a^**
22∶5n-6	13.3±2.1^a^	1.8±1.4^b^	4.3±2.8^a^	0.6±0.4^b^	0.4	8.2±1.1^b^	0.8±0.4^a^	6.7±0.7^b^	0.9±0.0^a^
Total n-6 PUFA	18.2±1.7^b^	24.2±3.6^a^	11.8±2.9	16.4±4.7	8.1	17.1±1.3	21.7±0.5	16.1±0.5^b^	20.6±0.2^a^
18∶3n-3	0.4±0.3^b^	1.3±0.4^a^	26.8±6.1	24.6±5.9	0.6	11.9±0.4	10.9±1.6	10.9±0.9	14.4±0.8
18∶4n-3	0.4±0.1^b^	0.8±0.1^a^	4.1±1.5	3.3±1.4	0.0	1.5±0.1^a^	0.8±0.1^b^	1.2±0.2	1.6±0.1
20∶5n-3	2.8±0.6^b^	7.4±2.5^a^	4.7±3.0	3.7±1.5	4.7	4.2±0.3^a^	2.1±0.1^b^	2.1±0.3^a^	1.6±0.0^b^
22∶5n-3	0.6±0.1	0.7±0.1	0.3±0.3	0.2±0.1	4.8	1.9±0.1	1.8±0.1	1.9±0.3	1.8±0.1
22∶6n-3	40.0±4.6^a^	5.3±0.9^b^	10.6±8.0	1.4±0.5	33.0	25.3±1.0^a^	8.7±0.8^b^	20.0±2.6^a^	6.1±0.7^b^
Total n-3 PUFA	46.0±4.0^a^	16.8±1.2^b^	46.4±3.6^a^	33.2±5.3^b^	43.1	45.4±1.5^a^	24.7±0.8^b^	36.7±3.9^a^	26.2±0.9^b^
Total PUFA	64.2±5.6^a^	41.0±4.3^b^	58.2±6.4^a^	49.6±1.0^b^	51.2	62.5±2.6	46.3±1.2	52.8±4.4^a^	46.8±0.9^b^
(n-3)/(n-6)	2.5±0.1^a^	0.7±0.1^b^	4.1±0.6^a^	2.3±1.1^b^	2.0	0.6±0.0^a^	0.3±0.0^b^	0.6±0.0^a^	0.3±0.0^b^
DHA/EPA	15.1±3.4^a^	0.9±0.6^b^	2.1±1.0^a^	0.4±0.2^b^	2.6	1.3±0.1	1.1±0.1	2.4±0.4^a^	0.8±0.1^b^
ARA/DHA	0.03±0.01^b^	2.0±0.4^a^	0.1±0.1^b^	4.7±2.0^a^	0.0	0.0±0.0^b^	0.3±0.1^a^	0.1±0.0^b^	0.4±0.0^a^
ARA/EPA	0.4±0.1^b^	1.6±0.7^a^	0.3±0.1^b^	1.8±0.5^a^	0.3	0.2±0.0^b^	1.4±0.1^a^	0.5±0.1^b^	1.4±0.1^a^
MUFA/PUFA	0.2±0.1^b^	1.0±0.2^a^	0.4±0.1^b^	0.7±0.1^a^	0.1	0.1±0.0^b^	0.2±0.0^a^	0.1±0.0^b^	0.1±0.0^a^

Totals include some minor components not shown. Superscript letters denote significant differences among diets for a given live prey or larval age (One-way ANOVA, *P*<0.05). A 0.0%TFA means content under 0.45%TFA.

* 100 g of ARA-H contained 29.0 g of Fluka© oil (Sigma-Aldrich, Chemie GmBH, Steinheim, Norway), 17.4 g of Vevodar® (DSM Food Specialties, Netherlands), 5.2 g of olive oil, 2.3 g of vitamin E, 4.1 g of soy lecithin and 42.0 g of distilled water.

Rotifers were enriched in 20 l containers at 500 rotifers ml^−1^ at 26°C with 0.6 g l^−1^ of each emulsion. Half of the rotifers were supplied to the larvae after 2 h of enrichment and the other half after 6 h of enrichment. One-day-old *Artemia* nauplii (EG strain, INVE) were enriched in 20 l containers at 300 nauplii ml^−1^ for 16h at 28°C with 0.6 g l^−1^ of each emulsion. Enriched *Artemia* were kept at 4°C in UV-treated, filtered seawater with aeration until administered to larvae twice a day. In order to reduce the bacterial load after enrichment and remove emulsion residues, rotifers and *Artemia* were washed with UV-treated filtered seawater and treated with H_2_O_2_ (40 ppm for 15 min for rotifers and 8000 ppm for 5 min for *Artemia,* according to [50]) and then rinsed with filtered seawater. The biochemical analysis of lipids and fatty acid (FA) composition [51] of enriched rotifer and *Artemia,* as well as in Senegalese sole larvae at 2, 15 and 50 dph are shown in Table 5.

For gene expression analyses, 200 mg wet weight pigmented juveniles and pseudo-albinos were sampled at 60 dph, sacrificed with an overdose of anesthetic (Tricaine methanesulfonate, MS-222, Sigma), rinsed in distilled water and preserved in RNAlater© (Ambion) at −80°C for further analyses.

### Metamorphosis

Eye migration was used as a measure of the progress of the metamorphosis process and consisted of evaluating the eye migration index (I_EM_) in 30 individuals per tank at 15, 30 and 50 dph according to [17]. Data were presented as the relative amount of larvae in each stage of development at the same age. The eye migration index (I_EM_ = Σ (%fish in each stage*stage)/100) was calculated according to [52].

### Photography

Larvae (at 22, 27, 33, 35 and 41 dph) and juveniles (at 60 dph) were photographed using a stereomicroscope (Nikon SMZ800, Soft Imaging Systems, GmbH) equipped with a Color View-XS camera. Skins of juveniles were photographed using a DP70 (Olympus) camera attached to DMLB (Leica) microscope. Images were taken under transmitted or incident light and compiled and processed using Analysis®3.1 (Soft Imaging Systems, GmbH, Germany). The amount of melanophores, xanthophores and iridophores in post-metamorphosed larvae was quantified in the ocular side of the trunk skin, excluding the abdominal area, using ImageJ64 software. Results were represented as the relative proportion, expressed in percentage, of each chromatophore in the analyzed skin area. The same software was used to measure, in µm, the size of chromatophores and the extension of dispersed melanin within melanophores in post-metamorphosed larvae.

### Partial cDNA Sequences Isolation

Several genes involved in the process of pigmentation were selected as markers for melanophore differentiation and melanin synthesis in Senegalese sole (Table 4). Very few Senegalese sole-specific gene sequences were available from public data bases, therefore multiple sequence homologs for the chosen target genes from extant species were downloaded from GenBank to construct alignments for use in designing primers homologous to conserved regions. Sequences were aligned using the CLUSTAL algorithm embedded in BioEdit ver 7.0.5.2 (T. Hall 1997–2005). Amplification was performed using a gradient thermal cycler (Eppendorf Mastercycler) to optimize specific amplification of target regions. The amplification products were separated and isolated by agarose gel electrophoresis for sequencing in direct sequencing reactions using both forward and reverse primers from the original amplification. The sequences obtained from amplification products were analyzed using BLAST to verify similitude to the intended target sequence. Genes used for expression studies in this work and their GenBank accession numbers are given (Table 4).

### Real Time PCR Assays

Gene expression in pigmented and pseudo-albino specimens was analyzed using the skin of 60 day-old juveniles (54±12.6 mg wet weight). Total RNA of a pool (5 individuals) of pigmented and a pool (5 individuals) of pseudo-albino specimens per each tank was extracted in separate using TRIzol™ (Invitrogen®, San Diego, CA, USA). The quantity of RNA isolated was determined using a Gene-Quant spectrophotometer (Amersham Biosciences). The quality of the RNA was examined using 1.2% agarose gel electrophoresis. Total RNA (1 µg) from each sample were reverse-transcribed using QuantiTect Reverse Transcription Kit (Qiagen®, GmbH, Germany). Genomic DNA was removed using genomic DNA wipe-out buffer included in the QuantiTect Reverse Transcription Kit. Selected samples were also run as non-RT negative controls to confirm the efficacy of the DNAse treatment. Real-time PCR analysis was performed using an ABI PRISM 7300 (Applied Biosystems). For each gene, a species-specific Taqman assay was designed (Applied Biosystems) (Table 4). Amplification reactions were performed in triplicate in a total volume of 20 µl containing 1 µl of cDNA, 1 µl of Taqman probe, 10 µl of Taqman mix and 8 µl of sterile water. The gene *ubq (ubiquitin)* was chosen as a reference gene since it did not exhibit any significant variation in expression among the samples [53]. The amplification conditions were 10 min at 95°C and 40 cycles of 15 s at 95°C and 1 min at 60°C.

Real-time PCR efficiencies were determined for each gene from the slopes obtained with Applied Biosystems software, applying the equation E = 10[−1/slope], where E is PCR efficiency. To determine the relative quantity of target gene-specific transcripts present in the different samples, expression ratios (R) were calculated according to the following formula: R = (E_target gene_) ^ΔCT target gene (mean sample - mean ref sample)/^(E*_ubq_*) ^ΔCT *ubq* (mean sample - mean ref sample)^, where *ubq* is the reference gene and mean sample corresponds to quadruplicate average. Pigmented individuals were chosen as ref samples.

### Statistics

Data are presented as mean ± SD (N = 4). Statistical analyses were conducted using SigmaStat 3.0 (Systat Software Inc., Richmond, USA). All data were checked for normality (Kolmogorov-Smirnov test) and homogeneity of variance (Bartlett’s test). A two-way ANOVA was performed to analyze the amount of chromatophores during development in both groups and a one-way ANOVA to analyze the amount of chromatophores of each group at each developmental date. When significant differences were found (*P*<0.05), the post-hoc Holm-Sidak method was used to perform all pairwise multiple comparisons. For the rest of the analyses, statistical significance was calculated using Student’s *t*-test (*P*<0.05).

## References

[1] Montoliu L, Oetting WS, Bennett DC (2011) Color Genes. European Society for Pigment Cell Research. Available: http://www.espcr.org/micemut Accessed 2013 Jun 10.

[2] Hofreiter M, Schöneberg T (2010) The genetic and evolutionary basis of colour variation in vertebrates. Cell Mol Life Sci 67: 2591–603. 20229234 10.1007/s00018-010-0333-7 PMC11115542

[3] Fujii R (2000) The Regulation of Motile Activity in Fish Chromatophores. Pigment Cell Res 13: 300–319. 11041206 10.1034/j.1600-0749.2000.130502.x

[4] Lister JA (2002) Development of pigment cells in the zebrafish embryo. Micros Res Tech 58: 435–441. 10.1002/jemt.10161 12242700

[5] Mellgrem EM, Johnson SL (2002) The evolution of morphological complexity in zebrafish stripes. Trends Genet 18: 128–134. 11858836 10.1016/s0168-9525(01)02614-2

[6] Patton EE, Mitchell DL, Nairn RS (2010) Genetic and environmental melanoma models in fish. Pigment Cell Melanoma Res 23: 314–337. 20230482 10.1111/j.1755-148X.2010.00693.x PMC2881310

[7] Kelsh RN, Brand M, Jiang YJ, Heisenberg CP, Lin S, et al (1996) Zebrafish pigmentation mutations and the processes of neural crest development. Development 123: 369–389. 9007256 10.1242/dev.123.1.369

[8] Parichy DM, Johnson SL (2001) Zebrafish hybrids suggest genetic mechanisms for pigment pattern diversification in Danio. Dev Genes Evol 211: 319–328. 11466528 10.1007/s004270100155

[9] Fukamachi S, Sugimoto M, Mitani H, Shima A (2004) Somatolactin selectively regulates proliferation and morphogenesis of neural-crest derived pigment cells in medaka. Proc Natl Acad Sci U S A 101: 10661–10666. 15249680 10.1073/pnas.0401278101 PMC489991

[10] Klovins J, Haitina T, Fridmanis D, Kilianova Z, Kapa I, et al (2004) The melanocortin system in Fugu: determination of POMC/AGRP/MCR gene repertoire and synteny, as well as pharmacology and anatomical distribution of the MCRs. Mol Biol Evol 21: 563–579. 14694081 10.1093/molbev/msh050

[11] Cerdá-Reverter JM, Ringholm A, Schiöth HB, Peter RE (2003) Molecular cloning, pharmacological characterization and brain mapping of the melanocortin 4 receptor in the goldfish: involvement in the control of food intake. Endocrinology 144: 2336–2349. 12746294 10.1210/en.2002-0213

[12] Cerdá-Reverter JM, Haitina T, Schiöth HB, Peter RE (2005) Gene structure of the goldfish agouti-signaling protein: a putative role in the dorsal-ventral pigment pattern of fish. Endocrinol 146: 1597–610. 10.1210/en.2004-1346 15591139

[13] Yamada T, Okauchi M, Araki K (2010) Origin of adult-type pigment cells forming the asymmetric pigment pattern, in Japanese flounder (*Paralichthys olivaceus*). Dev Dyn 239: 3147–62. 20941781 10.1002/dvdy.22440

[14] Guillot R, Ceinos RM, Cal R, Rotllant J, Cerdá-Reverter JM (2012) Transient ectopic overexpression of agouti-signalling protein 1 (*asip1*) induces pigment anomalies in flatfish. PLoS ONE 7(12): e48526. 23251332 10.1371/journal.pone.0048526 PMC3519472

[15] Darias MJ, Andree KB, Boglino A, Fernández I, Estévez A, et al (2013) Coordinated regulation of chromatophore differentiation and melanogenesis 1 during the ontogeny of skin pigmentation of *Solea senegalensis* (Kaup, 1858). PLoS ONE 8(5): e63005. 23671650 10.1371/journal.pone.0063005 PMC3650040

[16] Seikai T, Matsumoto J (1994) Mechanism of pseudoalbinism in flatfish: an association between pigment cell and skin differentiation. J World Aquac Soc 25: 78–85.

[17] Villalta M, Estévez A, Bransden MP (2005) Arachidonic acid enriched live prey induces albinism in Senegal sole (*Solea senegalensis*) larvae. Aquaculture 245: 193–209.

[18] Villalta M, Estèvez A, Bransden MP, Bell JG (2008) Arachidonic acid, arachidonic/eicosapentaenoic acid ratio, stearidonic acid and eicosanoids are involved in dietary-induced albinism in Senegal sole (*Solea senegalensis*). Aquac Nutr 14: 120–128.

[19] Lund I, Steenfeldt SJ, Hansen BW (2010) Influence of dietary arachidonic acid combined with light intensity and tank color on pigmentation of common sole (*Solea solea* L.) larvae. Aquaculture 308: 159–165.

[20] Kelsh RN (2004) Genetics and evolution of pigment patterns in fish. Pigment Cell Res. 17: 326–36. 10.1111/j.1600-0749.2004.00174.x 15250934

[21] Fernández-Díaz C, Yúfera M, Cañavate JP, Moyano FJ, Alarcón FJ, et al (2001) Growth and physiological changes during metamorphosis of Senegal sole reared in the laboratory. J Fish Biol 58: 1086–1097.

[22] Parichy DM, Turner JM (2003) Temporal and cellular requirements for Fms signaling during zebrafish adult pigment pattern development. Development 130: 817–833. 12538511 10.1242/dev.00307

[23] Nakamasu A, Takahashi G, Kanbe A, Kondo S (2009) Interactions between zebrafish pigment cells responsible for the generation of Turing patterns. Proc Natl Acad Sci U S A 106: 8429–34. 19433782 10.1073/pnas.0808622106 PMC2689028

[24] Fukamachi S, Yada T, Meyer A, Kinoshita M (2009) Effects of constitutive expression of somatolactin alpha on skin pigmentation in medaka. Gene 442: 81–7. 19393303 10.1016/j.gene.2009.04.010

[25] Lister JA, Robertson CP, Lepage T, Johnson SL, Raible DW (1999) Nacre encodes a zebrafish microphthalmia-related protein that regulates neural-crest-derived pigment cell fate. Development 126: 3757–67. 10433906 10.1242/dev.126.17.3757

[26] Minchin JE, Hughes SM (2008) Sequential actions of Pax3 and Pax7 drive xanthophore development in zebrafish neural crest. Dev Biol 317: 508–522. 18417109 10.1016/j.ydbio.2008.02.058 PMC3005709

[27] Kubic JD, Young KP, Plummer RS, Ludvik AE, Lang D (2008) Pigmentation PAX-ways, the role of Pax3 in melanogenesis, melanocyte stem cell maintenance, and disease. Pig Cell Mel Res 21: 627–45. 10.1111/j.1755-148X.2008.00514.x PMC2979299 18983540

[28] Galibert MD, Yavuzer U, Dexter TJ, Goding CR (1999) Pax3 and regulation of the melanocyte-specific tyrosinase-related protein-1 promoter. J Biol Chem 274: 26894–26900. 10480898 10.1074/jbc.274.38.26894

[29] Bondurand N, Pingault V, Goerich DE, Lemort N, Sock E, et al (2000) Interaction among *SOX10*, *PAX3* and *MITF*, three genes altered in Waardenburg síndrome. Hum Mol Genet 9: 1907–1917. 10942418 10.1093/hmg/9.13.1907

[30] Guo XL, Ruan HB, Li Y, Gao X, Li W (2010) Identification of a novel nonsense mutation on the Pax3 gene in ENU-derived white belly spotting mice and its genetic interaction with c-Kit. Pig Cell Mel Res 23: 252–62. 10.1111/j.1755-148X.2010.00677.x 20095975

[31] Hathaway JD, Haque A (2011) Insights into the Role of PAX-3 in the Development of Melanocytes and Melanoma. Open Cancer J 4: 1–6. 24790680 10.2174/1874079001104010001 PMC4002046

[32] Alexeev V, Yoon K (2006) Distinctive role of the cKit receptor tyrosine kinase signaling in mammalian melanocytes. J Invest Dermatol 126: 1102–10. 16410786 10.1038/sj.jid.5700125

[33] Curran K, Lister JA, Kunkel GR, Prendergast A, Parichy DM, et al (2010) Interplay between Foxd3 and Mitf regulates cell fate plasticity in the zebrafish neural crest. Dev Biol 344: 107–18. 20460180 10.1016/j.ydbio.2010.04.023 PMC2909359

[34] Levy C, Fisher DE (2011) Dual roles of lineage restricted transcription factors: The case of MITF in melanocytes. Transcription 2: 19–22. 21326905 10.4161/trns.2.1.13650 PMC3023642

[35] Aoki H, Moro O (2002) Involvement of microphthalmiaassociated transcription factor (MITF) in expression of human melanocortin-1 receptor (MC1R). Life Sci 71: 2171–2179. 12204775 10.1016/s0024-3205(02)01996-3

[36] Chen AS, Marsh DJ, Trumbauer ME, Frazier EG, Guan XM (2000) Inactivation of the mouse melanocortin-3 receptor results in increased fat mass and reduced lean body mass. Nat Genet 26: 97–102. 10973258 10.1038/79254

[37] Luo D, Chen H, Searles G, Jimbow K (1995) Coordinated mRNA expression of c-Kit with tyrosinase and TRP-1 in melanin pigmentation of normal and malignant human melanocytes and transient activation of tyrosinase by Kit/SCF-R. Melanoma Res 5: 303–9. 8541720 10.1097/00008390-199510000-00002

[38] Steingrimsson E, Copeland NG, Jenkins NA (2004) Melanocytes and the microphthalmia transcription factor network. Annu Rev Genet 38: 365–411. 15568981 10.1146/annurev.genet.38.072902.092717

[39] Ginger RS, Askew SE, Ogborne RM, Wilson S, Ferdinando D, et al (2008) SLC24A5 encodes a trans-Golgi network protein with potassium-dependent sodium-calcium exchange activity that regulates human epidermal melanogenesis. J Biol Chem 283: 5486–95. 18166528 10.1074/jbc.M707521200

[40] Lamason RL, Mohideen MA, Mest JR, Wong AC, Norton HL, et al (2005) SLC24A5, a putative cation exchanger, affects pigmentation in zebrafish and humans. Science 310: 1782–6. 16357253 10.1126/science.1116238

[41] Vogel P, Read RW, Vance RB, Platt KA, Troughton K, et al (2008) Ocular albinism and hypopigmentation defects in Slc24a5−/− mice. Vet Pathol 45: 264–79. 18424845 10.1354/vp.45-2-264

[42] Schallreuter KU, Kothari S, Chavan B, Spencer JD (2008) Regulation of melanogenesis–controversies and new concepts. Exp Dermatol 17: 395–404. 18177348 10.1111/j.1600-0625.2007.00675.x

[43] Nadeau NJ, Minvielle, Ito S, Inoue-Murayama M, Gourichon D, et al (2008) Characterization of Japanese quail yellow as a genomic deletion upstream of the avian homolog of the mammalian ASIP (agouti) gene. Genetics 178: 777–86. 18287407 10.1534/genetics.107.077073 PMC2248353

[44] Sviderskaya EV, Hill SP, Balachandar D, Barsh GS, Bennett DC (2001) Agouti signaling protein and other factors modulating differentiation and proliferation of immortal melanoblasts. Dev Dyn 221: 373–9. 11500974 10.1002/dvdy.1153

[45] Hunt G, Kyne S, Wakamatsu K, Ito S, Thody AJ (1995) Nle^4^DPhe^7^∝-melanocyte stimulating hormone increases the eumelanin: Pheomelanin ratio in cultured human melanocytes. J Invest Dermatol 104: 83–85. 7798647 10.1111/1523-1747.ep12613565

[46] Uong A, Zon LI (2010) Melanocytes in development and cancer. Cell Physiol. 222: 38–41. 10.1002/jcp.21935 PMC2783760 19795394

[47] Logan DW, Bryson-Richardson RJ, Pagan KE, Taylor MS, Currie PD, et al (2003) The structure and evolution of the melanocortin and MCH receptors in fish and mammals. Genomics 81: 184–191. 12620396 10.1016/s0888-7543(02)00037-x

[48] Kilkenny C, Browne WJ, Cuthill IC, Emerson M, Altman DG (2010) Improving Bioscience Research Reporting: The ARRIVE Guidelines for Reporting Animal Research. PLoS Biol 8(6): e1000412. 20613859 10.1371/journal.pbio.1000412 PMC2893951

[49] Cañavate JP, Zerolo R, Fernández-Díaz C (2006) Feeding and development of Senegal sole (*Solea senegalensis*) larvae reared in different photoperiods. Aquaculture 258: 368–377.

[50] Giménez G, Padro F, Roque A, Estévez A, Furones D (2006) Bacterial load reduction of live prey for fish larval feeding using Ox-Aquaculture. Aquac Res 37: 1130–1139.

[51] Boglino A, Darias MJ, Ortiz-Delgado JB, Özcan F, Estévez A, et al (2012) Commercial products for Artemia enrichment affect growth performance, digestive system maturation, ossification and incidence of skeletal deformities in Senegalese sole (*Solea senegalensis*) larvae. Aquaculture 324–325: 290–302.

[52] Solbakken JS, Norberg B, Watanabe K, Pittman K (1999) Thyroxine as a mediator of metamorphosis of Atlantic halibut, *Hippoglossus hippoglossus* . Environ Biol Fish 56: 53–65.

[53] Manchado M, Infante C, Asensio E, Cañavate JP, Douglas SE (2007) Comparative sequence analysis of the complete set of 40S ribosomal proteins in the Senegalese sole (S*olea senegalensis* Kaup) and Atlantic halibut (*Hippoglossus hippoglossus* L.) (Teleostei: Pleuronectiformes): phylogeny and tissue-and development-specific expression. BMC Evol Biol 7: 107. 17608926 10.1186/1471-2148-7-107 PMC1933418

